# Metastatic Urothelial Carcinoma With Sarcomatoid Subtype After Robot-Assisted Radical Cystectomy Successfully Treated With Pembrolizumab

**DOI:** 10.7759/cureus.61871

**Published:** 2024-06-07

**Authors:** Kazumasa Murase, Keita Nakane, Makoto Kawase, Koji Iinuma, Takuya Koie

**Affiliations:** 1 Urology, Gifu University Graduate School of Medicine, Gifu, JPN; 2 Urology, Gifu University, Gifu, JPN

**Keywords:** pembrolizumab, sarcomatoid subtype, metastatic urothelial carcinoma, robot-assisted radical cystectomy, muscle-invasive bladder cancer

## Abstract

A 76-year-old man who was diagnosed with urothelial carcinoma (UC) in the bladder diverticulum was referred to our institution. The patient was diagnosed with muscle-invasive bladder cancer, which was confirmed by magnetic resonance imaging that showed tumor invasion into the fatty tissue surrounding the diverticulum. After two cycles of neoadjuvant gemcitabine and cisplatin, he underwent robot-assisted radical cystectomy (RARC) with pelvic lymph node dissection followed by intracorporeal ileal conduit. The histopathologic diagnosis of the bladder tumor was UC with squamous differentiation and sarcomatoid subtype and ypT3bN0M0 without positive surgical margins. The patient refused any adjuvant therapy. Six months after RARC, the patient visited our institution with a complaint of suddenly occurring generalized pain. Because ^18^F-fluorodeoxyglucose positron emission tomography-CT showed multiple metastases, including bone, para-aortic lymph nodes, and pleura, pembrolizumab was initiated as a second-line treatment. After two courses of pembrolizumab, the patient's symptoms remarkably improved, and the abnormal systemic accumulation on PET-CT almost disappeared. After 26 months of continuous treatment with pembrolizumab, the patient remains disease-free. Several studies have been reported that focused on tumor subtypes and programmed cell death ligand 1 (PD-L1)-positive tumor cells as candidate biomarkers in relation to the efficacy of pembrolizumab. The higher proportion of PD-L1-positive cells in the sarcomatoid subtype may have resulted in favorable oncological outcomes compared with pure UC.

## Introduction

Muscle-invasive bladder cancer (MIBC) with sarcomatoid subtype has a poorer prognosis than those with pure urothelial carcinoma (UC) alone [1]. Pembrolizumab is the immune checkpoint inhibitor used as second-line therapy and has been shown to be effective in locally advanced or metastatic UC that has progressed during or after treatment with platinum-based combination chemotherapy [2]. We report a case of a patient with postoperative recurrence of MIBC, including sarcomatoid subtype, who underwent robot-assisted radical cystectomy (RARC) after cisplatin-based neoadjuvant therapy (NAC), who received pembrolizumab as second-line therapy and had a significant response. Although pembrolizumab has been administered continuously for 26 months, the patient has maintained no evidence of disease.

## Case presentation

**Figure 1:**
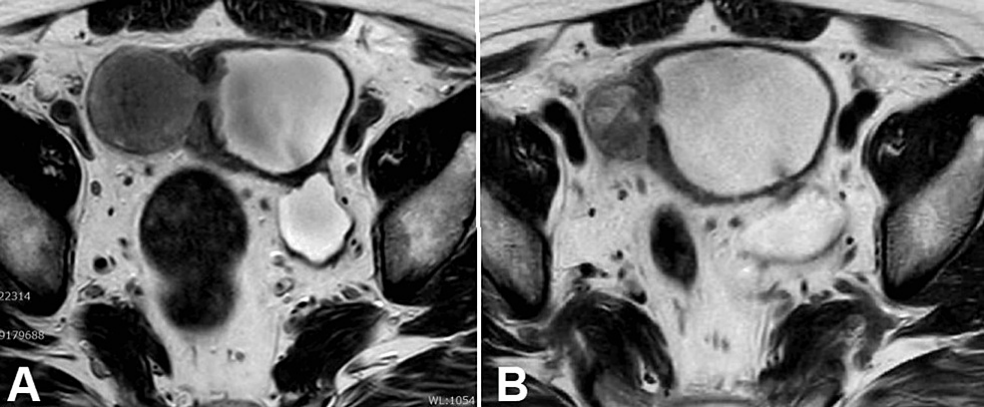
The status of bladder tumors before and after neoadjuvant chemotherapy. Magnetic resonance imaging showed a bladder tumor within a bladder diverticulum. (A) Tumor before platinum-based neoadjuvant chemotherapy. (B) After two cycles of platinum-based neoadjuvant chemotherapy, the tumor was found to have decreased in size by more than 50%.

**Figure 2:**
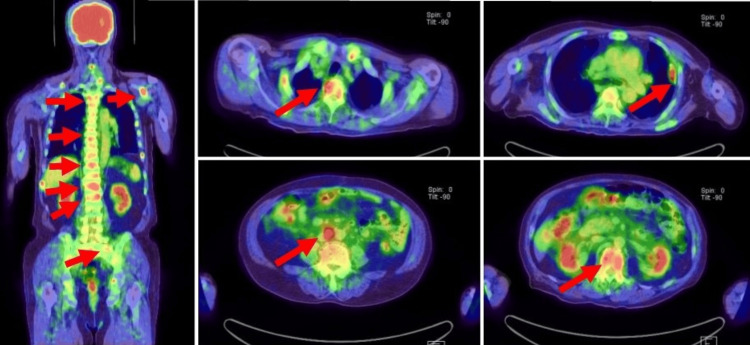
Evaluation of multiple metastases using 18F-fluorodeoxyglucose positron emission tomography-computed tomography. Six months after robot-assisted radical cystectomy, ^18^F-fluorodeoxyglucose positron emission tomography-computed tomography showed multiple bone metastases, para-aortic lymph node metastases, and pleural metastases (red arrowheads).

**Figure 3:**
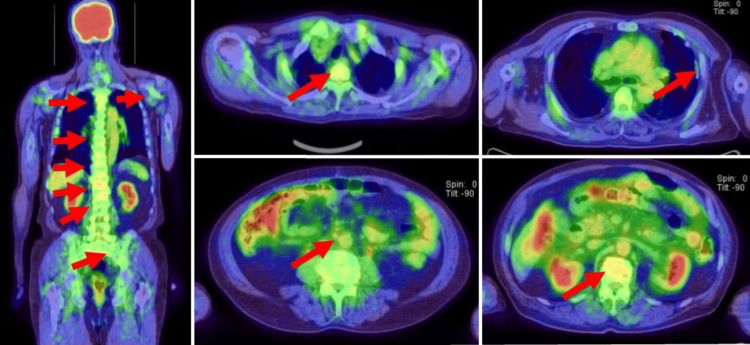
The status of multiple metastases after the administration of pembrolizumab. After two courses of pembrolizumab, the patient's symptoms remarkably improved and the abnormal systemic accumulation on ^18^F-fluorodeoxyglucose positron emission tomography-computed tomography almost disappeared (red arrowheads).

A 76-year-old man who was diagnosed with UC in the bladder diverticulum was referred to our institution for further examination and subsequent treatment. The tumor filled the bladder diverticulum and could not be completely resected by transurethral resection of bladder tumor (TURBT). The pathological diagnosis was invasive UC; however, the invasion into the fatty tissue surrounding the bladder diverticulum was unclear. Thoracoabdominal and pelvic computed tomography (CT) and magnetic resonance imaging (MRI) revealed that the patient was suggestive of being diagnosed with MIBC at cT3aN0M0 (Figure 1). After two cycles of platinum-based NAC, CT and MRI showed that the bladder tumor had decreased in size by more than 50% (Figure 2B). Thereafter, he underwent RARC with pelvic lymph node dissection followed by intracorporeal ileal conduit. The histopathologic diagnosis of the bladder tumor was UC, ypT3pN0M0 with squamous differentiation, and sarcomatoid subtype. Although the tumor identified lymphovascular invasion, the resection margins were negative. The patient refused any adjuvant therapy, including immune checkpoint inhibitors. Six months after the surgery, the patient visited our institution with a complaint of suddenly occurring generalized pain. Based on ^18^F-fluorodeoxyglucose positron emission tomography-CT (^18^F-FDG PET-CT) showing multiple bone metastases, para-aortic lymph node metastases, and pleural metastases (Figure 2), pembrolizumab was initiated as a second-line treatment. After two courses of pembrolizumab, the patient's symptoms remarkably improved and the abnormal systemic accumulation on PET-CT almost disappeared (Figure 3). Grade 1 erythema and diarrhea were the most common adverse events associated with pembrolizumab; however, no other serious immune-related adverse events were observed during the treatment period. Seven months after initiation of pembrolizumab, the patient developed lymph node metastasis at the hepatis portal region, which resolved with local radiotherapy. Although pembrolizumab has been administered continuously for 26 months since the initiation of second-line treatment, the patient remains disease-free.

## Discussion

Pembrolizumab is a humanized monoclonal antibody against programmed cell death 1 (PD-1), and blockade of the PD-1-programmed cell death ligand 1 (PD-L1) interaction has the potential to be an effective approach to tumor-specific immunotherapy [2]. The KEYNOTE-045 trial evaluated the efficacy of pembrolizumab as second-line therapy in patients with UC who had progressed after platinum-based chemotherapy [2]. In this study, the median overall survival (OS) for patients who received pembrolizumab was 10.3 months, compared to 7.4 months for those who received chemotherapy, with significantly longer OS in the pembrolizumab group. The proportion of patients who received pembrolizumab who had a response duration of at least 12 months was approximately two-fold higher than that of patients who received chemotherapy, and furthermore, subgroup analysis showed the efficacy of pembrolizumab compared to chemotherapy [2]. Based on the results of this trial, pembrolizumab became covered by national health insurance in Japan for chemotherapy-refractory unresectable UC in 2017.

In the KEYNOTE045 study, approximately 70% of enrolled patients had pure UC, while the remaining 30% contained histologic subtypes [2]. MIBC with sarcomatoid subtype, as in the present case, is considered a unique type, accounting for 0.1-0.3% of all cases, with more cases already advanced in stage at diagnosis compared to pure UC [1]. Several studies have been reported that focused on tumor subtypes and PD-L1-positive tumor cells as biomarkers in relation to the efficacy of pembrolizumab [3,4]. The microarray study using tissue from 11,838 different tumor samples reported that 29.2% of samples with pure pT2-T4 UC contained ≥10% PD-L1-positive cells, compared to 70.8% for the sarcomatoid subtype of UC [3]. Kobayashi et al. [4] investigated the treatment effect of pembrolizumab for each histological subtype of UC and performed a propensity score matching analysis. The median OS for patients with the sarcomatoid subtype and pure UC was not reached and 7.8 months, respectively, indicating a significantly better prognosis for those with the sarcomatoid subtype [4]. The higher proportion of PD-L1-positive cells in the sarcomatoid subtype may have resulted in favorable oncological outcomes compared with pure UC [4]. In addition, a meta-analysis of the efficacy of pembrolizumab in UC refractory to platinum-based chemotherapy reported that an Eastern Cooperative Oncology Group Performance Status of ≥2 correlated with OS [5]. In this case, extensive metastases with generalized pain were observed six months after surgery, suggesting that long-term survival may have been achieved because the patient had UC with a sarcomatoid subtype and pembrolizumab treatment was initiated immediately.

## Conclusions

We report a case of postoperative recurrence of MIBC with sarcomatoid subtype that was treated with pembrolizumab as second-line therapy and maintained in long-term remission. The results suggested that oncologic outcomes may be improved in patients with MIBC having sarcomatoid subtype with a high risk of disease progression if the timing of pembrolizumab initiation is not missed.
